# RNA is an Adjuvanticity Mediator for the Lipid-Based Mucosal Adjuvant, Endocine

**DOI:** 10.1038/srep29165

**Published:** 2016-07-04

**Authors:** Masayuki Hayashi, Taiki Aoshi, Koji Ozasa, Takato Kusakabe, Masatoshi Momota, Yasunari Haseda, Shingo Kobari, Etsushi Kuroda, Kouji Kobiyama, Cevayir Coban, Ken J. Ishii

**Affiliations:** 1 Laboratory of Adjuvant Innovation, National Institute of Biomedical Innovation, Health and Nutrition, Osaka 567-0085, Japan; 2 Laboratory of Vaccine Science, WPI Immunology Frontier Research Center, Osaka University, Osaka 565-0871, Japan; 3 Vaccine Research Development Office, Biology Research Laboratories, Sohyaku. Innovative Research Division, Mitsubishi Tanabe Pharma Corporation, Kanagawa 227-0033, Japan; 4 Department of Pediatrics, Yokohama City University Graduate School of Medicine, Kanagawa 236-0004, Japan; 5 Laboratory of Malaria Immunology, WPI Immunology Frontier Research Center, Osaka University, Osaka 565-0871, Japan

## Abstract

Nasal vaccination has the potential to elicit systemic and mucosal immunity against pathogens. However, split and subunit vaccines lack potency at stimulating mucosal immunity, and an adjuvant is indispensable for eliciting potent mucosal immune response to nasal vaccines. Endocine, a lipid-based mucosal adjuvant, potentiates both systemic and mucosal immune responses. Although Endocine has shown efficacy and tolerability in animal and clinical studies, its mechanism of action remains unknown. It has been reported recently that endogenous danger signals are essential for the effects of some adjuvants such as alum or MF59. However, the contribution of danger signals to the adjuvanticity of Endocine has not been explored. Here, we show that RNA is likely to be an important mediator for the adjuvanticity of Endocine. Administration of Endocine generated nucleic acids release, and activated dendritic cells (DCs) in draining lymph nodes *in vivo*. These results suggest the possibility that Endocine indirectly activates DCs via damage-associated molecular patterns. Moreover, the adjuvanticity of Endocine disappeared in mice lacking TANK-binding kinase 1 (*Tbk1*), which is a downstream molecule of nucleic acid sensing signal pathway. Furthermore, co-administration of RNase A reduced the adjuvanticity of Endocine. These data suggest that RNA is important for the adjuvanticity of Endocine.

Natural infections with respiratory viruses such as influenza virus strongly elicit systemic and mucosal immune responses, and these immune responses prevent re-infection. The secretory IgA mucosal antibody in particular has been shown to possess neutralization activity against hetero-subtype viral strains and prevent viral entry into host cells by neutralizing invading viruses at their port of entry^1^,^2^. However, parenteral vaccination barely induces a mucosal immune response. Therefore, to improve a vaccine’s ability to prevent viral entry and improve vaccine cross-reactivity by eliciting mucosal immunity, various nasal vaccines have been developed and tested in preclinical or clinical studies^3^,^4^, but the exact mechanism of action for most of them (except TLR ligands) is still unknown.

Most adjuvants target pathogen recognition receptors (PRRs) such as Toll-like receptors (TLRs), RIG-like receptors, Nod-like receptors (NLRs), C-type lectin receptors (CLRs), AIM2-like receptors (ALRs) or cytoplasmic DNA sensors, and PRR engagement triggers expression of co-stimulatory molecules on antigen presenting cells, inflammatory cytokines and type I interferon (IFN) production via downstream molecules^5^,^6^. Furthermore, it has been reported that some adjuvants cause cell damage at the administration site and indirectly activate innate immunity via the damage-associated molecular pattern (DAMP) molecules released from damaged cells. Thus far, various molecules, such as the high-mobility group box 1 (HMGB1), nucleic acids or ATP, have been identified as DAMPs^7^. Among these DAMPs, ATP and DNA are key molecules for the adjuvanticity of MF59 and alum, respectively^8^,^9^.

Endocine, a novel mucosal adjuvant consisting of oleic acid and mono-olein, has developed for nasal vaccines and has been shown in animal studies and clinical trials to be robustly immunogenic with the tolerability^10^,^11^. However, the mechanism of action of Endocine remains unknown. In this study, we found that DAMPs are likely to be required for the adjuvanticity of Endocine.

## Results

### Endocine induces both systemic and mucosal immune responses

**Figure 1:**
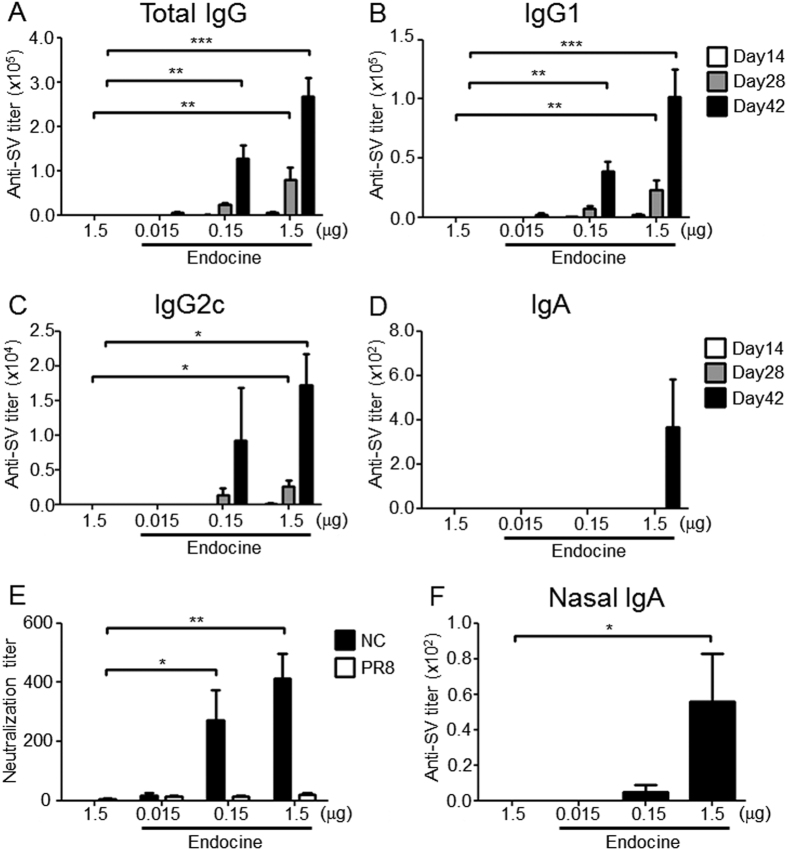
Endocine potentiates both systemic and mucosal antibody responses. C57BL/6j mice (n = 4) were immunized three times intranasally (days 0, 14, 28) with SV alone or together with 2% Endocine. SV-specific (**A**) total IgG, (**B**) IgG1, (**C**) IgG2c and (**D**) IgA titers in sera at days 14, 28 and 42, and (**F**) IgA titer in nasal wash samples at day 42 were measured by ELISA. (**E**) Viral neutralization titer in sera at day 42 was measured by a microneutralization assay. The results represent three separate experiments. Median and SEM are shown for each group. The statistically significant values (*P < 0.05, **P < 0.01, ***P < 0.001) shown were obtained from Dunnett’s multiple comparison tests.

The adjuvanticity of Endocine has been shown in BALB/c mice and ferrets^11^,^12^, but its adjuvanticity in C57BL/6 mice has not been evaluated. To confirm the adjuvanticity conferred by Endocine on the influenza splitHA vaccine (SV) in C57BL/6 mice, Endocine-adjuvanted SV at 0.015 to 1.5 μg was immunized i.n. three times at two-weekly intervals. Then, the antigen-specific antibody responses in mouse sera and nasal washes were examined. As shown in Fig. 1A, Endocine generated significant SV-specific antibody responses at 0.15 μg of SV, whereas no antibody responses were observed at 1.5 μg of SV alone. Additionally, analysis of the antibody subclasses showed that Endocine elicited a Th2 type subclass, IgG1-biased antibody response (Fig. 1B–D). Furthermore, the virus neutralization assay showed that SV-specific antibodies in sera generated by Endocine administration significantly neutralized the influenza virus (Fig. 1E). Notably, Endocine also elicited SV-specific IgA production in the nasal washes, whereas no IgA response was observed in SV alone group (Fig. 1F), thus demonstrating that Endocine potentiated the systemic and mucosal immune responses in the C57BL/6 mice.

### TLR2 and TLR4 are not required for the adjuvanticity of Endocine

**Figure 2:**
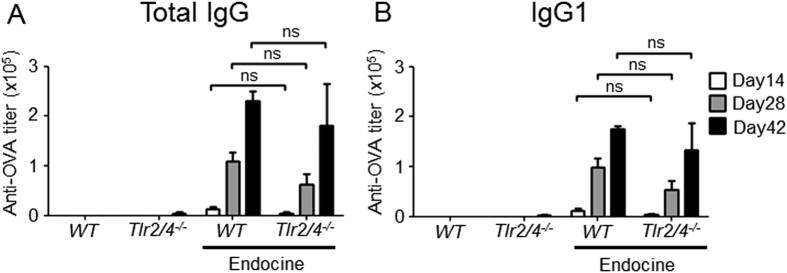
TLR2 and TLR4 are not required for the adjuvanticity of Endocine. Both *Tlr2*- and *Tlr4*-deficient mice (n = 4) were immunized three times intranasally (days 0, 14, 28) with 10 μg of OVA protein alone or together with 2% Endocine. The OVA-specific (**A**) total IgG and (**B**) IgG1 titers in sera at days 14, 28 or 42 were measured by ELISA. The results represent two separate experiments. Median and SEM are shown for each group. Statistically significant values are indicated, ns: not significant by Student’s *t*-test.

Although the present study and previous reports have shown that Endocine, a lipid-based adjuvant, is a promising mucosal vaccine adjuvant^10^,^11^,^12^, its mechanism of action is still unknown. Some lipid adjuvants such as monophosphoryl lipid A and glucopyranosyl lipid (GLA) are known to potentiate adjuvanticity via TLR4^13^,^14^. Therefore, to investigate the mechanism of action of Endocine, we first examined its adjuvanticity in C57BL/6 background mice lacking both *Tlr2* and *Tlr4*, which recognize lipoprotein and some lipids^6^,^15^. Endocine-adjuvanted OVA was administered i.n. to the *Tlr2/4*^*−/−*^ mice and the resultant antibody responses were evaluated. As shown in Fig. 2A,B, a similar antibody response was seen in the *Tlr2/4*-deficient mice as was observed in the wild type mice. This result shows that TLR2 and TLR4 are not required for the adjuvanticity of Endocine.

### Endocine generates DAMPs release following cell damage

**Figure 3:**
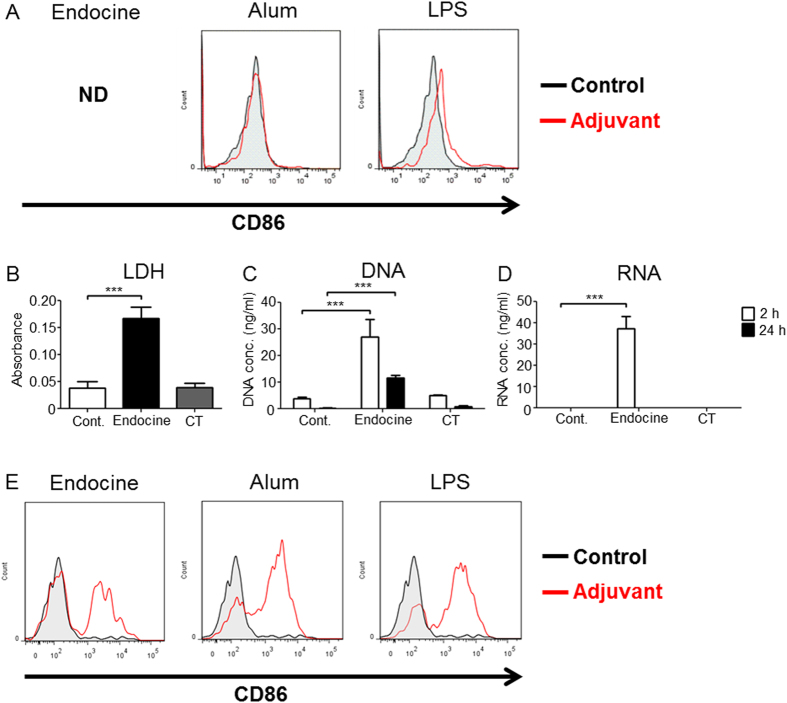
Endocine generates DAMPs release following cell damage. (**A**) Mouse bone marrow derived FL-DCs were stimulated with 0.1% Endocine, alum (1 mg/ml) or LPS (50 ng/ml) for 24 h, and CD86 expression on pDCs was analyzed by FACS. (**B**) Nasal washes were collected 2 h after nasal administration of 2% Endocine or CT (n = 5). After the cells in the fluids were removed by centrifugation, LDH activities in the supernatants were measured. (**C,D**) Nasal washes were collected 2 or 24 h after nasal administration of 2% Endocine or CT (n = 5). After the cells in the fluids were removed by centrifugation, the DNA and RNA concentrations in the supernatants were measured. (**E**) After administering 2% Endocine, 0.67 mg alum or 50 ng LPS to the base of the tail, CD86 expression on pDCs in inguinal lymph nodes was analyzed by FACS. The results represent two or three separate experiments. Median and SEM are shown for each group. ND: Not Detected. Statistical significances indicated as ***P < 0.001 were obtained from Dunnett’s multiple comparison test or Student’s t-test.

Because DCs play a central role in adjuvant-induced immune responses, the ability of Endocine to activate DCs was examined. Mouse bone marrow-derived DCs were stimulated with Endocine, alum or LPS *in vitro*, and the activation marker CD86 expression on DCs was analyzed by FACS. Alum stimulation did not activate DCs, whereas LPS stimulation resulted in DC activation (Fig. 3A). In the case of Endocine, a very low frequency of live cells was detected by FACS, implying that Endocine decreased cell viability. Therefore, to investigate if Endocine causes cytotoxicity, epithelial cell, A549 cells were treated with Endocine and LDH activity released from damaged cells was examined. As expected, Endocine treatment showed cytotoxicity in epithelial cells *in vitro* (Supplemental Fig. S1A,B). Furthermore, LDH activity in nasal wash samples after i.n. administration of Endocine was significantly increased (Fig. 3B), suggesting Endocine causes cell damage *in vivo*. These results gave a speculation that the DAMPs released from the damaged cells contributed to the adjuvanticity of Endocine.

Some adjuvants, such as alum or MF59, cause cell damage at the injection site, generating DAMPs release from the damaged cells. These DAMPs activate the innate immune response through PRRs^16^. Therefore, to investigate whether Endocine generates DAMPs release from damaged cells *in vivo*, extracellular nucleic acid, which is a DAMP molecule, was examined in the nasal wash samples after nasal administration of Endocine. Nucleic acid concentration in the nasal washes was measured 2 or 24 h after Endocine administration (Fig. 3C,D). As expected, extracellular DNA and RNA were detected in mice administered with Endocine, whereas cholera toxin (CT), a nasal adjuvant used here as a benchmark, did not cause these DAMPs release. Therefore, we hypothesized that Endocine functions as an adjuvant, at least partly, through the activation of innate immunity by DAMP molecules. To examine whether Endocine indirectly activates DCs *in vivo* via DAMPs, DC activation in draining lymph nodes was analyzed by FACS after Endocine or alum were administered to mice. Interestingly, Endocine activated DCs *in vivo* (Fig. 3E). Additionally, alum also increased CD86 expression on DCs *in vivo*, whereas alum did not affect CD86 expression on DCs *in vitro* (Fig. 3A,E). As it has been shown that extracellular DNA released from damaged cells is important for alum adjuvanticity^9^, this adjuvant possibly indirectly activates DCs via DNA *in vivo*. These results suggest that DAMPs are important for the adjuvanticity of Endocine.

### Endocine adjuvanticity is independent of the NLR pyrin domain-containing 3 (NLRP3) inflammasome

**Figure 4:**
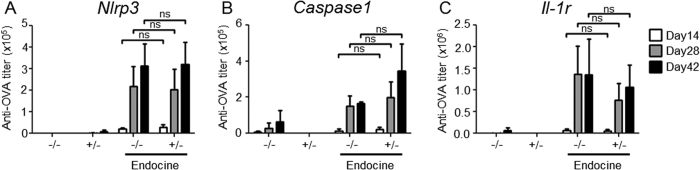
The NLRP3 inflammasome is independent of the adjuvanticity of Endocine. (**A**) *Nlrp3*^*−/−*^ or ^+/−^, (**B**) *Caspase1*^*−/−*^ or ^+/−^, or (**C**) *Il-1r*^*−/−*^ or ^+/−^ mice (n = 3) were immunized three times intranasally (days 0, 14, 28) with 10 μg OVA alone or together with 2% Endocine. The OVA-specific total IgG titer in sera at days 14, 28 or 42 was measured by ELISA. The results represent two separate experiments. Median and SEM are shown for each group. Statistically significant values are indicated, ns: not significant by Student’s *t*-test.

NLRP3, a member of the NLR family, is a component of the inflammasome and senses various types of pathogens or irritants such as *Candida albicans*, *Legionella pneumophila*, alum and silica^17^,^18^,^19^,^20^. Sensing these pathogens induces proinflammatory cytokine, IL-1β production through caspase-1 activation. Additionally, it has been reported recently that NLRP3 senses not only pathogens but also some endogenous DAMP molecules such as ATP or uric acid^21^,^22^,^23^,^24^,^25^. Thus, to investigate whether the NLRP3 inflammasome is involved in the adjuvanticity of Endocine, OVA-specific antibody responses in mice lacking *Nlrp3*, *Caspase1* or *Il-1r* were examined after nasal administration of OVA mixed with Endocine. The antibody response was not affected by the absence of *Nlrp3*, *Caspase1* or *Il-1r*, suggesting that the NLRP3 inflammasome pathway is not required for the adjuvanticity of Endocine (Fig. 4).

### Nucleic acids are important for the adjuvanticity of Endocine

**Figure 5:**
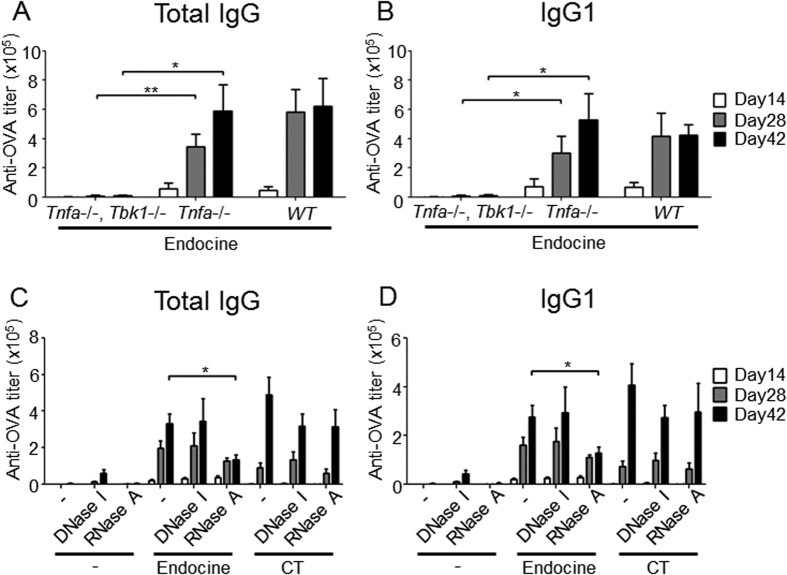
Nucleic acids are required for the adjuvanticity of Endocine. C57BL/6j mice (WT), *Tnfa*^*−/−*^ or *Tnfa*^*−/−*^*, Tbk1*^*−/−*^ mice (n = 3) were immunized three times intranasally (days 0, 14, 28) with 10 μg of OVA alone or together with 2% Endocine. OVA-specific (**A**) total IgG and (**B**) IgG1 titers in sera at days 14, 28 or 42 were measured by ELISA. DNase I or RNase A was co-administered three times intranasally (days 0, 14, 28) with Endocine or CT-adjuvanted OVA to the C57BL/6j mice (n = 5). OVA-specific (**C**) total IgG and (**D**) IgG1 titers in sera at days 14, 28 or 42 were measured by ELISA. The results represent two separate experiments. Median and SEM are shown for each group. Statistically significant values (*P < 0.05, **P < 0.01) were obtained by Student’s *t*-tests.

Our data showed that Endocine generates DNA and RNA release at the vaccine administration site (Fig. 3C,D). Thus, to investigate nucleic acid involvement in the adjuvanticity of Endocine, mice lacking *Tbk1*, which is a downstream molecule in the nucleic acids sensing signaling pathway^26^, in a *Tnfa*-deficient background were used because *Tbk1*^*−/−*^ mice die in utero, and this lethal effect can be rescued in the absence of *Tnfa*^27^. Endocine-adjuvanted OVA was administered i.n. to *Tbk1*^*−/−*^ mice, and the OVA-specific antibody responses were analyzed in such mice. Interestingly, Endocine lost its adjuvanticity in the absence of *Tbk1* (Fig. 5A,B), suggesting that extracellular nucleic acids are essential for its adjuvanticity. It has been reported that hydroxypropyl-β-cyclodextrin (HP-β-CD) and alum exert their adjuvant properties via triggering extracellular DNA release in which the adjuvanticity was diminished by co-administration of DNase, suggesting that extracellular DNA is an important adjuvant mediator^28^,^29^. Therefore, to further identify if DNA and RNA are both essential for the adjuvanticity of Endocine, DNase I or RNase A was co-administered with Endocine-adjuvanted OVA to the mice and their immunogenicity was evaluated. Intriguingly, RNase A, but not DNase I, significantly reduced the adjuvanticity of Endocine, whereas RNase A did not significantly influence the adjuvanticity of CT (Fig. 5C,D). Moreover, to confirm the activity of co-administered DNase I or RNase A *in vivo*, the concentration of DNA and RNA in the nasal washes were examined after i.n. co-administration of DNase I or RNase A with Endocine. RNA in the nasal washes was clearly disappeared by RNase A co-administration, but conversely, DNase I did not reduce the concentration of DNA (Supplemental Fig. S2A,B). Due to difficulties to perform DNase I treatment in smaller volume for i.n. administration, we used additional approach to test the contribution of DNA to the adjuvanticity of Endocine. Cytosolic DNA sensor, stimulator of interferon gene (*Sting*)^30^ mutant (Goldenticket, Tmem173^gt^) mice, which have a loss-of-function mutation at the ligand-binding site of STING^31^, were utilized for further experiments. OVA-specific antibody response was analyzed in *Sting* mutant mice after i.n. administration of Endocine-adjuvanted OVA. Loss-of-function of STING did not have influence on the adjuvanticity of Endocine, suggesting that cytosolic DNA sensing machinery through STING is not required for the adjuvanticity of Endocine (Supplemental Fig. S3). Collectively, our findings suggest that nucleic acid, especially RNA, is one of the mediators of Endocine adjuvanticity.

### The adjuvant effect of Endocine is independent of the canonical nucleic acid sensing machinery

**Figure 6:**
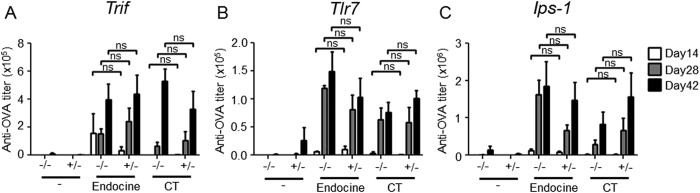
Canonical RNA sensors are independent of the adjuvanticity of Endocine. (**A**) *Trif*^*−/−*^ or ^+/−^ (n = 4–5), (**B**) *Tlr7*^*−/−*^ or ^+/−^ (n = 3–5), or (**C**) *Ips-1*^*−/−*^ or ^+/−^ (n = 5) mice were immunized three times intranasally (days 0, 14, 28) with 10 μg of OVA alone or together with 2% Endocine or CT. The OVA-specific total IgG titer in sera at day 14, 28 and 42 was measured by ELISA. Statistically significant values are indicated, ns: not significant by Student’s *t*-test.

Finally, we attempted to identify the RNA sensing receptor required for the adjuvanticity of Endocine. TLR3 preferentially senses double-stranded RNA (dsRNA) derived from some viruses or host messenger RNA^32^,^33^,^34^. On the other hand, TLR7 responds to single-stranded RNA^35^. While these TLRs act as RNA receptor at endosome, members of RLR family, retinoic acid-inducible gene I (RIG-I) and melanoma differentiation-associated protein 5 (MDA5) recognize short RNA and long dsRNA in cytoplasm, respectively^26^. In addition, these TLRs and RLRs trigger type I IFN response through the activation of interferon regulatory factor (IRF) 3/7^36^. To examine the involvement of these canonical RNA sensors and type I IFN pathway in the adjuvanticity of Endocine, we analyzed antigen-specific antibody responses in mice lacking the following canonical RNA sensing machinery-related genes: *Trif* (which encodes TIR-domain-containing adapter-inducing interferon-β, an adaptor molecule for TLR3), *Tlr7* (which encodes TLR7), and *Ips-1* (which encodes interferon-β promoter stimulator I, an adaptor molecule for RIG-I and MDA5), and type I IFN regulatory transcription genes: *Irf3* and *Irf7*. The mice were examined after administration of Endocine-adjuvanted OVA. However, the adjuvanticity of Endocine was unaffected in mice lacking *Trif*, *Tlr7, Ips-1*, *Irf3* and *Irf7* genes (Fig. 6A–C and Supplemental Fig. S4). These results demonstrate that, at least in mice, the adjuvanticity of Endocine is independent of the canonical RNA sensors TLR3, TLR7 and RIG-I, and type I IFN pathway.

## Discussion

Activation of innate immunity is indispensable for the induction of adaptive immune responses. Like CpG-oligodeoxynucleotides or LPS, TLR ligands induce an adaptive immune response following activation of innate immunity via engagement with TLRs on DCs. In addition to pathogen-associated molecular patterns (PAMPs) such as TLR ligands, it has been recently reported that alum or MF59 activates innate immunity via the DAMPs released from damaged cells^8^,^9^. The present study has shown that DAMPs are important for the adjuvanticity of Endocine. In particular, extracellular RNA seems to be one of the essential mediators for Endocine adjuvanticity. On the other hand, extracellular DNA or ATP is required for the adjuvanticity of alum or MF59, respectively^8^,^9^. However, it remains unknown why different DAMPs are required for the adjuvanticity of such adjuvants. The kind of DAMPs released from damaged cells might differ depending on the type of cell damage or cell death (e.g., apoptosis, necrosis, necroptosis or pyroptosis). How Endocine induces cell damage, or what type of cell death it generates, has not been determined. Endocine induces temporary DNA/RNA release 2 h after its administration, whereas alum induces DNA release 24 h after it is administered (Fig. 3C,D and Supplemental Fig. S5). This implies that Endocine induces cell damage by a different mechanism than that of alum, or at least with a different time kinetic. Indeed, there are interesting reports that chronic aluminum exposure induces oxidative DNA damage^37^, and oxidized mitochondrial DNA activates the NLRP3 inflammasome, inducing subsequent IL-1β production^38^. These reports prompt speculation that the quality of the DNA released (e.g., oxidized DNA) might be different depending on the type of cell damage or cell death. Although we extensively searched by *in vitro* and *in vivo* (using *Sting* mutant mice) assays, however, we could not conclude the contribution of DNA to the adjuvanticity of Endocine (Fig. 3C and Supplemental Fig. S2A). Co-administration of DNase has been shown to decrease the adjuvanticity of HP-β-CD or alum immunized by s.c. or i.m. route, respectively^28^,^29^. In contrast to these reports, smaller amount of DNase was used in our study because larger volume cannot be utilized for i.n. vaccination without entering into lung. Since nasal vaccine in human is not delivered to the lung, large volume administration delivered to lung may result in misinterpretation of nasal vaccine evaluation. Although we utilized i.n. administrable maximum amount of DNase, it might not be adequate for the digestion of DNA generated by Endocine administration. To further clarify the contribution of DNA for the adjuvanticity of Endocine, we utilized *Sting* mutant mice. Our results showed that loss of function of STING did not influence on the adjuvanticity of Endocine (Supplemental Fig. S3). Therefore, at least, cytosolic DNA sensor, STING is not required for the adjuvanticity of Endocine, but the exact necessity of extracellular DNA for the adjuvanticity of Endocine remained to be examined in future.

Endocine consists of surface-active lipids; therefore, it probably also works as a delivery system, transporting antigens through the mucus in the nose. Hence, it most probably works as both a delivery system and an adjuvant. The adjuvanticity of other lipid-based adjuvants such as cationic N3OA, which is composed of oleylamine, and cationic N3OASq, which is composed of oleylamine and squalene, has been compared with Endocine in a previous study^11^. In that study, Endocine and N3OA induced higher antibody responses than N3OASq, while the highest cellular immune response was induced by N3OASq. To understand the mechanism of action of DAMPs as mediators of adjuvanticity, it is important to analyze why different immune responses are induced by these lipid-based adjuvants.

In summary, this study identifies RNA as a DAMP signal for the adjuvanticity of Endocine. To identify the RNA recognition receptor required for the adjuvanticity of Endocine, we also investigated the involvement of the canonical RNA sensing machinery including *Trif*, *Tlr7*, and *Ips-1*. Unfortunately, none of these single gene deficient mice showed reduced adjuvanticity of Endocine (Fig. 6). We were unable to identify the receptor or likely pathway for RNA activation in this study. However, we found that TBK1 is essential for the adjuvanticity of Endocine (Fig. 5A,B), strongly suggesting that tissue damage-released nucleic acids (DNA or RNA) are involved in the adjuvanticity of Endocine. TBK1 can be activated via multiple pathways^39^. Sensing of RNA generated by Endocine administration may be operated via more than one already known pathways or involvement of novel RNA recognition machinery may yet exist in addition to currently known mechanisms. Involvement of DNA for the adjuvanticity of Endocine was inconclusive in this study, and remains to be examined in future research.

## Methods

### Mice

Six-week-old female C57BL/6j mice were purchased from CLEA Japan. *Tlr2/4*^*−/−*^, *Trif*^*−/−*^ and *Tlr7*^*−/−*^ mice were purchased from Oriental BioService Inc., Japan. *Il-1r*^*−/−*^ mice were purchased from The Jackson Laboratory. Generation of *Ips-1*^*−/−*^, *Nlrp3*^*−/−*^, *Caspase1*^*−/−*^, *Sting*^*Gt/Gt*^ (Tmem173^gt^), *Irf3*^*−/−*^, *Irf7*^*−/−*^, *Tnfa*^*−/−*^ and *Tbk1*^*−/−*^ mice have been described previously^28^,^40^,^41^,^42^. All animal experimental protocols were approved by the Institutional Animal Care and Use Committee at the Japanese National Institute of Biomedical Innovation, and all animal experiments were performed in accordance with the Institutional Guidelines for the Japanese National Institute of Biomedical Innovation, Health and Nutrition Animal Facility.

### Antigens, adjuvants and peptides

Ovalbumin was purchased from Invivogen. The SV derived from the influenza A/New Caledonia/20/99 strain was gifted from the Institute of Microbial Chemistry. Alum was purchased from Sigma. Endocine was from Eurocine Vaccines AB. Cholera toxin (CT) and cholera toxin B subunit (CTB) were purchased from Wako.

### Nasal immunizations

Mice were immunized three times intranasally (i.n.) with 2-week intervals (days 0, 14, 28). Blood samples were taken on days 14, 28 and 42. During vaccination and bleeding, the mice were anesthetized with ketamine. Nasal washings were collected by flushing the nasal passages with 1 ml of PBS. Endocine was administrated at 2%. The CT adjuvant was prepared by mixing 1 ng of CT with 1 μg of CTB per mouse. DNase I (Roche) or RNase A (Sigma) was used at 400 or 40 μg/mouse, respectively. All nasal administration volumes were 5 μl per nostril.

### Antigen-specific Ab titers

For the SV-vaccinated group, 96-well plates were coated with 1 μg/ml SV in a carbonate buffer (pH 9.6), or 10 μg/ml OVA for the OVA-vaccinated group. The wells were blocked with 1% BSA in PBS, and diluted sera from the vaccinated mice were incubated on the antigen-coated plate. After washing, either goat anti-mouse total IgG, IgG1 or IgG2c conjugated HRP (Southern Biotech) were added and incubated for 1 h at room temperature. After additional washing, the plates were incubated with TMB (KPL) for 30 min, the reaction was stopped with 2 N H_2_SO_4_, and the absorbance was measured with an OD_450_ of 0.2 as the cut-off value for positive samples.

### Microneutralization titers

Sera from the immunized mice were mixed with receptor-destroying enzyme II (Denka-Seiken) and then incubated overnight at 37 °C. After further incubation for 1 h at 56 °C, the serially diluted sera at a final titer of 100 tissue culture infectious dose_50_/ml of A/New Caledonia/20/1999 (NC) (H1N1) or A/Puerto Rico/8/34 (PR8) (H1N1) influenza viruses in MEM media containing 10 mM HEPES, 1% penicillin-streptomycin (Nacalai Tesque), 0.2% BSA and 10 μg/mL trypsin were incubated for 30 min at 37 °C. Next, the viruses were added to Madin-Darby Canine kidney (MDCK) cells. Four days after incubation at 37 °C with 5% CO_2_, the MDCK cells were fixed with 10% formalin for 10 min at room temperature. The cells were stained with naphthol blue black solution (naphthol blue black 0.5 g, sodium acetate 0.5 g, acetic acid 45 ml and distilled water 455 ml) for 30 min at room temperature. The stained cells were washed thoroughly with water and then air-dried. Subsequently, 0.1 M NaOH was added to the cells, and the plates were read at 630 nm in a microplate reader. The virus neutralization titer was determined from the maximum dilution ratio, which gave a higher absorbance value than the averages of the positive and negative controls.

### *In vitro* cytotoxicity assay

2.5 × 10^4^ cells of lung epithelial cell, A549 cells (ATCC) were plated on 96 well plates, incubated for 15 h and treated with Endocine at indicated concentrations or 1% Triton X-100. After the treatments, LDH activities in supernatants were measured by Cytotoxicity Detection Kit ^plus^ (LDH) (Roche). When LDH activity from 1% Triton X-100-treated cells was set as maximum cytotoxicity (100%), the cytotoxicity from Endocine-treated cells was shown by the ratio of LDH activity to the maximum.

### Measuring the LDH activity and DNA/RNA concentration in nasal wash samples

Nasal washes were collected 2 or 24 h after i.n. administration of 10 μl of 2% Endocine or CT (5 μl per nostril) together with DNase I or RNase A. After the cells in the fluids were removed by centrifugation, the LDH activities in the supernatants were measured by Cytotoxicity Detection Kit ^plus^ (LDH) (Roche) and the cytotoxicity was presented by the absorbance (A_492nm_–A_620nm_). The concentrations of DNA and RNA were measured by a Qubit 2.0 Fluorometer (Life Technologies).

### DC activation *in vitro* and *in vivo*

For the *in vitro* experiment, bone marrow-derived DCs were generated by cultivation of bone marrow cells in RPMI 1640 supplemented with 10% FBS, 1% penicillin/streptomycin solution (Nacalai Tesque) and 100 ng/ml of human fms-like tyrosine kinase 3 ligand (Flt3L) (PeproTech) for 7 days. Flt3L-induced DCs (FL-DCs), stimulated for 15 h with 1 mg/ml alum, 0.1% Endocine or 50 ng/ml LPS (Sigma), were evaluated for CD86 expression on plasmacytoid DCs (pDCs) by FACS. CD11c^+^/SiglecH^+^ cells defined as pDCs were used for analysis.

*In vivo* experiments were performed as described previously^43^. Briefly, mice were injected with either 0.67 mg alum, 2% Endocine or 50 ng LPS to the base of the tail. Inguinal lymph nodes (iLNs) were collected from the mice 24 h after the administration of either adjuvant. To prepare single-cell suspensions, the iLNs were treated with DNase I (0.1 mg/ml) and collagenase D (1 mg/ml) for 30 min at 37 °C. Cells prepared by this way were stained with anti-mCD11c (HL3), -mPDCA-1 (JF05-1C2.4.1), -mCD86 (GL1) antibodies and 7-amino-actinomycin, and then analyzed by FACS. CD11c^+^/mPDCA-1^+^ cells defined as pDCs were used for analysis.

### Statistical analysis

Statistical significance (P < 0.05) between groups was determined by Dunnett’s multiple comparison test or Student’s *t*-test.

## Additional Information

**How to cite this article**: Hayashi, M. *et al*. RNA is an Adjuvanticity Mediator for the Lipid-Based Mucosal Adjuvant, Endocine. *Sci. Rep*. **6**, 29165; doi: 10.1038/srep29165 (2016).

## Supplementary Material

Supplementary Information
