# Supplementary material for: RNA is an Adjuvanticity Mediator for the Lipid-Based Mucosal Adjuvant, Endocine
Source: Sci Rep. 2016 Jul 4;6:29165. doi: 10.1038/srep29165 (PMC4931589; doi:10.1038/srep29165)
Supplement: Supplementary Information [file srep29165-s1.pdf]

RNA is an Adjuvanticity Mediator for the Lipid-Based Mucosal Adjuvant, Endocine

Running title: DAMPs mediates adjuvanticity of Endocine

Masayuki Hayashi,[1,2,3] Taiki Aoshi,[1,2,‖] Koji Ozasa,[1,2,4] Takato Kusakabe,[1,2] Masatoshi Momota,[1,2] Yasunari Haseda,[1,2,‖] Shingo Kobari,[1,2,4] Etsushi Kuroda,[1,2] Kouji Kobiyama,[1,2,#] Cevayir Coban,[5] and Ken J. Ishii,[1,2,*]

[1]Laboratory of Adjuvant Innovation, National Institute of Biomedical Innovation, Health and Nutrition, Osaka 567-0085, Japan
[2]Laboratory of Vaccine Science, WPI Immunology Frontier Research Center, Osaka University, Osaka 565-0871, Japan
[3]Vaccine Research Development Office, Biology Research Laboratories, Sohyaku. Innovative Research Division, Mitsubishi Tanabe Pharma Corporation, Kanagawa 227-0033, Japan
[4]Department of Pediatrics, Yokohama City University Graduate School of Medicine, Kanagawa 236-0004, Japan
[5]Laboratory of Malaria Immunology, WPI Immunology Frontier Research Center, Osaka University, Osaka 565-0871, Japan

Current address
[‖]Vaccine Dynamics Project, BIKEN Innovative Vaccine Research Alliance Laboratories, Research Institute for Microbial Diseases (RIMD), Osaka University, Osaka 565-0871, Japan
[#]La Jolla Institute for Allergy and Immunology, La Jolla, CA, USA

*Corresponding author:
Ken J Ishii, M.D., Ph.D.
Laboratory of Adjuvant Innovation, National Institute of Biomedical Innovation, Health and Nutrition (NIBIOHN)
7-8-5 Saito-Asagi, Ibaraki, Osaka 567-0085, Japan.
Tel: +81-72-641-8043; Fax: +81-72-641-8079;
E-mail: kenishii@biken.osaka-u.ac.jp, kenishii@nibiohn.go.jp

Supplemental Fig. S1

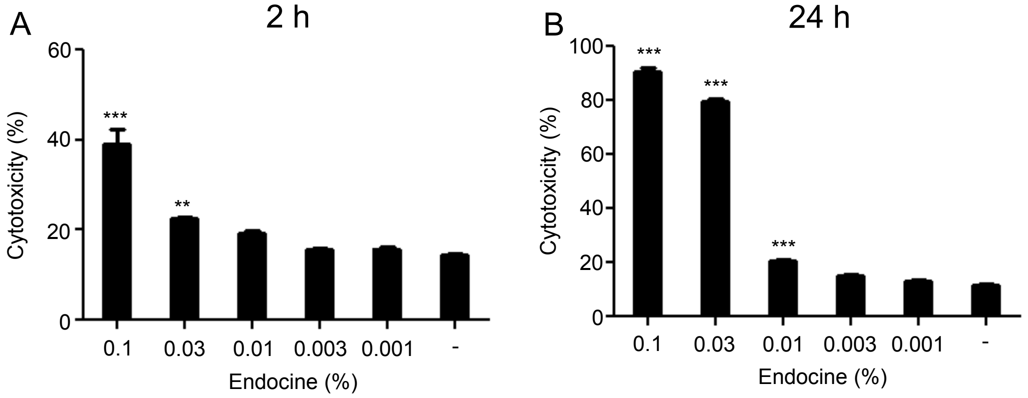

Supplemental Fig. S1. Endocine causes epithelial cell damages *in vitro*. A549 cells were treated with Endocine for (A) 2 or (B) 24 h at the concentration indicated, and the LDH activity in supernatants was measured. The cytotoxicity was calculated by the ratio of LDH activity to 1% Triton X-100-treated cells. The results were presented as the median and SEM of five wells. Statistically significant values, indicated as **P<0.01, ***P<0.001, were obtained from Dunnett's multiple comparison test.

Supplemental Fig. S2

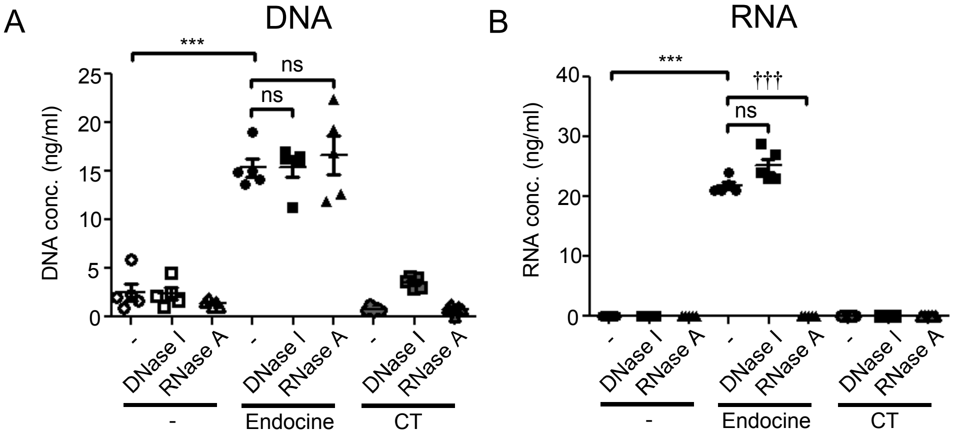

Supplemental Fig. S2. Extracellular RNA disappears by co-administration of RNase A. Endocine or CT was i.n. co-administrated with DNase I or RNase A. Two hour after the administration, nasal washes were collected, and concentration of (A) DNA or (B) RNA in the fluids was measured. The statistically significant values (****P*<0.001, †††*P*<0.001, ns: not significant) shown were obtained from Dunnett's multiple comparison test or Student's *t*-test.

Supplemental Fig. S3

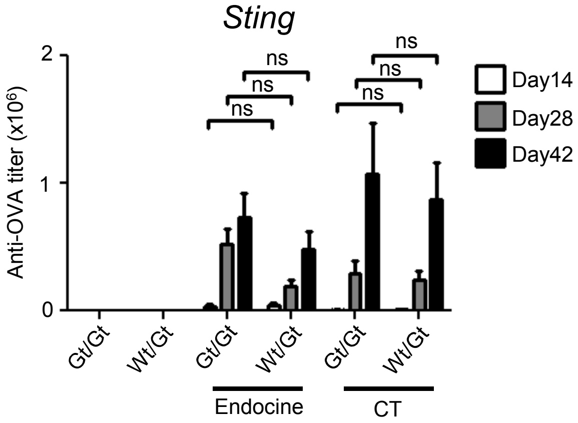

Supplemental Fig. S3. Cytosolic DNA sensor, STING is independent of the adjuvanticity of Endocine. *Sting*$^{Gt/Gt}$ or $^{Wt/Gt}$ mice (n=4-6) were immunized three times intranasally (day 0, 14, 28) with 10 μg of OVA alone or together with 2% Endocine or CT. The OVA-specific total IgG titer in sera at day 14, 28 and 42 was measured by ELISA. Statistically significant values are indicated, ns: not significant by Student's *t*-test.

Supplemental Fig. S4

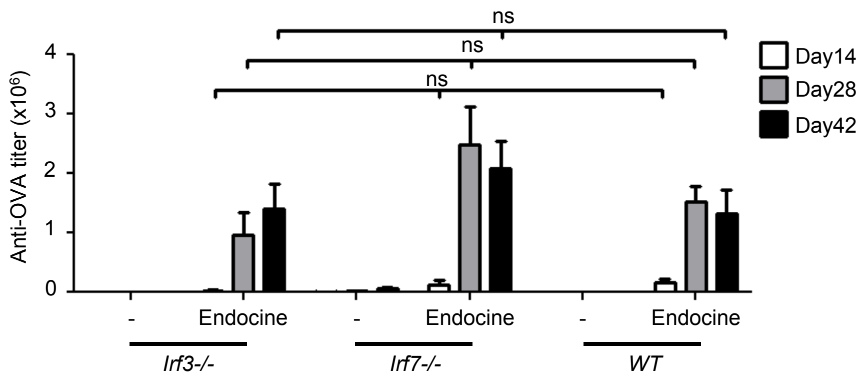

Supplemental Fig. S4. Type I IFN regulatory transcription genes: IRF3 and IRF7 are not required for the adjuvanticity of Endocine. *Irf3*$^{-/-}$, *Irf7*$^{-/-}$ or *WT* mice (n=3) were immunized three times intranasally (day 0, 14, 28) with 10 μg of OVA alone or together with 2% Endocine. The OVA-specific total IgG titer in sera at day 14, 28 and 42 was measured by ELISA. Statistically significant values are indicated, ns: not significant by Student's *t*-test.

Supplemental Fig. S5

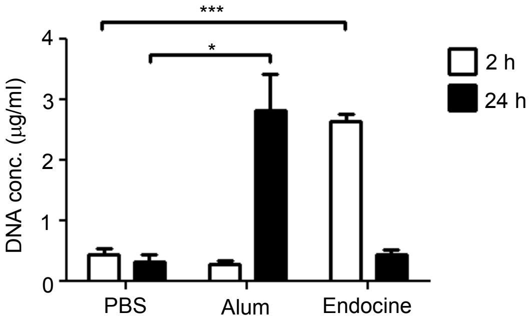

Supplemental Fig. S5. Alum generates DNA release 24 hours after administration. C57BL/6j mice (n=3) were i.p. administered 0.67 mg of alum, 500 µg (2%) of Endocine, or PBS. Peritoneal lavage fluids were collected 2 or 24 hours after administration of each adjuvant. After the cells in the fluids were removed by centrifugation, the DNA concentrations in the supernatants were measured. The results represent three separate experiments. Median and SEM are shown for each group. Statistically significant values, indicated as *P<0.05 and ***P<0.001, were obtained from Student's t-tests.
